# Social and Mental Health Factors Involved in the Severity of Loneliness in Older Individuals in a Spanish Rural Area

**DOI:** 10.3390/nursrep14040273

**Published:** 2024-11-29

**Authors:** Mayra Alejandra Mafla-España, Silvia Corchón, Paula Jimeno-de Pedro, Vanessa Ibáñez-del Valle, Omar Cauli

**Affiliations:** 1 Department of Nursing, Faculty of Nursing and Podiatry, University of Valencia, C/de Méndez y Pelayo, 19, 46010 Valencia, Spain; maymaes@alumni.uv.es (M.A.M.-E.); silvia.corchon@uv.es (S.C.); omar.cauli@uv.es (O.C.); 2 Frailty Research Organized Group (FROG), University of Valencia, 46010 Valencia, Spain; 3 Colisée Fundation, 46015 Valencia, Spain; redrural@fundacioncolisee.es; 4 Chair of Active Ageing, University of Valencia, 46010 Valencia, Spain

**Keywords:** loneliness, older people, rural area, social factors, nursing care plans

## Abstract

**Background**: Loneliness in older people, especially those living in rural areas, is a phenomenon that has received little attention in research and can have detrimental effects on quality of life. The aim of this study was to evaluate loneliness and the psychosocial factors associated with loneliness in rural Spain, which have been minimally studied. **Methods**: A cross-sectional study was carried out in a sample of permanently resident older people in the *Rincón de Ademuz* region (Valencia, Spain), a geographic area with very low population density. Emotional and social loneliness were assessed using the de Jong Gierveld Loneliness Scale. We also assessed whether loneliness is associated with sleep quality, depressive symptoms, and autonomy in basic and instrumental activities of daily living. **Results**: A total of 108 community-dwelling individuals aged 65 years and older participated in the study out of a total sample of 181. Of them, 30.6% experienced feelings of moderate loneliness, while 2.8% presented severe loneliness. A significant correlation was found between loneliness and age (Rho = 0.28, *p* = 0.003). Significant differences were also observed between emotional loneliness and gender (*p* = 0.03) but not between social loneliness and the total score on the de Jong Gierveld Scale. Men experienced more emotional loneliness than women. In the multivariate analyses, significant associations were found between the degree of loneliness and having sons/daughters (*p* = 0.03; odds ratio [OR] = 0.24; 95% CI 0.06–0.89) and the role of caring for a dependent person (*p* = 0.002; odds ratio [OR] = 0.05; 95% CI 0.009–0.36) but not living with sons/daughters or the presence of grandchildren. **Conclusions**: There is a high prevalence of loneliness among older people living in rural areas, which is associated with some social factors. Therefore, nursing care plans should include assessments and interventions to prevent or detect and address loneliness in older people. This study was retrospectively registered in ClinicalTrials on 24 April 2024 with registration number NCT06382181.

## 1. Introduction

It is estimated that more than 2.5 million elderly people in Spain experience feelings of loneliness, which amounts to almost 40% of the population over 65 years of age. Approximately 28.8% of older women and 14.7% of men live alone. Loneliness is generally defined as the discrepancy between a person’s desired level of social contact and a person’s actual level of interaction [1,2,3]. According to studies, up to 40% of adults over 65 years of age admit to feeling lonely at least occasionally, and this is more common at older ages, i.e., beyond 70 years of age [4]. Importantly, loneliness refers to perceived social isolation rather than objective social isolation [5,6,7]. Peplau and Perlman describe it as an unpleasant experience characterized by the painful feeling of social isolation that accompanies the perception of deficiencies in the quantity or quality of social relationships [8]. Perceptions play a crucial role in this definition; one person can have few social relationships and not feel lonely, while another can have many relationships and still experience loneliness. Loneliness is, therefore, more related to the perception of the quality of social relationships than to the quantity [4,5].

A conceptual review based on qualitative studies on loneliness identified three distinct yet interrelated types of conceptualizations: social, emotional, and existential loneliness [9]. Social loneliness is considered an “objective” condition determined by a few social connections that generate a feeling of disconnection from others. Meanwhile, emotional loneliness refers to the perception of social isolation and the lack of quality relationships [6,7]. The third type, existential loneliness (EL), is less commonly described. Unlike the other two types, EL not only involves the absence of meaningful relationships but also a sense of being fundamentally separated from others and the world at large [9].

Loneliness is a universal experience that has been shown to have detrimental effects on the health of older people [10]. Feelings of loneliness have serious consequences for health outcomes [11]. Prospective studies have shown that loneliness predicts depressive symptoms [12], as well as mental health and cognition problems [13], as well as admission to nursing homes [14]. Furthermore, loneliness is a powerful risk factor for suicidal ideation [15] and alcoholism [16]. Previous research on loneliness has suggested that it is related to faster aging and physiological decline [5], as well as mortality in older adults [7,17,18,19,20]. The influences of social isolation, both objective and subjective, on mortality risk are comparable to well-established mortality risk factors [7,20]. Several environmental and lifestyle factors, such as smoking, sedentary lifestyle, and air pollution, are considered risk factors for premature mortality [20]. However, much less attention has been paid in the scientific literature to social factors that have been shown to have an equivalent or greater influence on mortality risk [21]. Being socially connected not only influences psychological and emotional well-being but also has a significant and positive influence on physical well-being [22] and overall longevity [19,20,21,23].

Older individuals often face chronic health problems and life events, such as being a widow/widower or children living away from home, that make loneliness more likely in the elderly; thus, they may live in social isolation. [24,25]. It has been suggested that the association between age and loneliness is due to fewer opportunities for social contact due to health problems and age-related functional limitations or to age- or health-related reductions in social roles that previously offered opportunities for social contact (e.g., retirement or widowhood) [11]. Poor health and physical limitations are associated with increased loneliness. Racial/ethnic differences in loneliness are postulated to be due to the disadvantaged position of minority populations in key social and economic aspects. Low levels of education and income are associated with higher levels of loneliness in older black and Hispanic adults [26].

There are other psychosocial risk factors that lead to loneliness. Loneliness can differ depending on social roles [27]. Some of these factors have been studied, such as gender and marital status; for example, being married ensures at least one social connection, which is generally relatively strong in terms of protection against loneliness, while the loss of attachment relationships can increase it [28]. The stresses of unemployment, inadequate financial resources, and marital or family conflict have been associated with increased loneliness, as has the role of caring for dependent people [11]. Along with aging, the chronic stress experienced by caregivers of people with dementia often leads to deteriorating health. Studies have shown that spouse caregivers of people with Alzheimer’s disease report significantly higher levels of loneliness and depression than non-caregivers [29]. Another study showed that older caregivers of people with dementia reported significantly more perceived stress, depressive symptomatology, and loneliness than non-caregivers of a similar age, education, global cognition, and functional independence [30]. Older adults who care for dependent people in their homes are particularly vulnerable to the negative effects of chronic stress on emotional and physical health. To date, there has been a paucity of studies exploring the potential impact of geographic factors on individuals’ loneliness [31,32]. One gap identified related to this aspect is whether there are differences between living in rural and urban areas in terms of loneliness [33]. On one side, it could be considered that rural areas might be associated with greater loneliness due to the long distances, lack of infrastructure and transportation, and sparse populations of such regions [31,34]. On the other hand, there are usually stronger community connections in rural areas, which could be advantageous in reducing social isolation and loneliness [33,35]. Indeed, research findings regarding urban and rural differences in social isolation and loneliness have been inconsistent [33,35], suggesting the need for further research on these possible differences. In Spain, as in other places, the dynamics of social networks of older adults in rural communities and their relationship with loneliness have not been studied.

Spain is currently one of the European countries that suffers most from depopulation of rural areas, with 52% of the population living in cities of more than 50,000 inhabitants, while only 3.2% live in municipalities with fewer than 1000 inhabitants, a phenomenon currently called “Emptied Spain” [36]. Among the depopulated rural areas, *Rincón de Ademuz* is the least populated region of Valencia (located 2 h northwest of Valencia (driving)), with a population density of 6 inhabitants per square kilometer, which places it among the most unpopulated areas in Spain. In addition, in recent decades, it has experienced a significant demographic recession, which has stabilized in recent years. Among its characteristics, it can be highlighted that it has an aging population with low density, high dispersion, and negative growth. This rural area of the Valencian Community has suffered significant depopulation coupled with a marked socioeconomic crisis linked to its rural location, lack of industry, and few services [37]. Therefore, we hypothesized that the population could perceive social isolation and loneliness, and we considered it imperative to explore this issue and associated social and mental health factors.

Very few studies have addressed the problem of loneliness among older people residing in rural areas. Therefore, our objective was to investigate the prevalence and factors associated with loneliness in older people residing in the rural area of *Rincón de Ademuz*. We assessed whether loneliness is associated with demographic, social, and health characteristics, as well as the influences of the presence of family members, depressive symptoms, sleep quality, and basic and instrumental activities of daily living on the experience of loneliness in this specific context.

## 2. Materials and Methods

### 2.1. Study Design and Population

The study was carried out using a cross-sectional and observational design to understand the prevalence and factors associated with loneliness among older people living in the rural area of *Rincón de Ademuz*. This research was carried out by the Chair of Healthy, Active, and Participatory Ageing of the University of Valencia, in collaboration with the Colisée Foundation.

The study included 108 participants, both men and women, selected from the population of interest. To be eligible, participants had to be 65 years old or older, reside in the *Rincón de Ademuz* region, be autonomous in performing basic activities of daily living, and have good cognitive and verbal performance (as judged by a psychologist) to understand and respond to the questionnaire used in the study. The sample was selected first by contacting the mayors’ offices of the 7 municipalities and the health center of the *Rincón de Ademuz* region. These entities distributed information brochures about the study to older individuals who met the inclusion requirements (being 65 years of age or older and not suffering from cognitive impairment). Interested persons left their telephone numbers to be first contacted by phone; then, if they agreed, the psychologist went to their homes to present the study, obtain a signature for the informed consent form, and conduct the interview. Based on estimation of municipalities’ data, in the municipalities of the *Rincón de Ademuz* region, 652 people over 65 years of age were counted, but those living permanently (year-round) in the region numbered about 280 people (the rest usually live most of the year in different cities close to their children). Of these, those living in the community and not suffering from cognitive impairment were estimated to total 156. Accepting an alpha risk of 0.95 for a precision of +/− 0.05 units in a two-sided test for an estimated proportion of 35% of loneliness, 108 subjects (out 156) were considered sufficient for the target population. However, the sampling procedure was based on convenience and participants’ availability, and it was not randomized.

The estimation of loneliness a priori (35%) was based on a pilot study—more specifically, data published reporting a prevalence of around 30–40% in rural areas of Spain [38]. Considering the final results of our study, we observed a prevalence of 30.6% of loneliness in the study sample, leading to a sample size of 106 participants, which fits with the a priori estimation based on the literature.

The research protocol was approved by the Research Ethics Committee of the University of Valencia (code: UV-INV_ETICA-2013703; approval date: 10 January 2022), ensuring compliance with ethical principles and protection of the rights of the participants. All the participants signed the informed consent form. The study was conducted in accordance with the ethical principles outlined in the Declaration of Helsinki and the Belmont Report. All participants signed a consent form whereby they were informed of the terms of their participation and the study research goals. Data were anonymized, and only the research team had access to them. The confidentiality of the participants’ personal data was guaranteed throughout the entire research process. Participants were also informed that they could discontinue their participation at any time without further consequences.

### 2.2. Information Collection Procedure

An evaluation instrument composed of the de Jong Gierveld Loneliness Scale 2010 specifically designed to measure both emotional and social loneliness was used for the information collection process. This scale, validated in Spanish in previous studies [39], was implemented to evaluate the degree of loneliness experienced by the participants.

In addition to applying the Loneliness Scale, sociodemographic data were collected through the use of a questionnaire designed for this purpose. This questionnaire addressed aspects such as age, gender, marital status, and whether the participant lived with other people, among other factors relevant to the study of loneliness in older people.

The data were collected through individual interviews conducted by a trained psychologist, who was responsible for explaining the purpose of the study and guiding the participants in the process of completing the evaluation instruments. The confidentiality and privacy of the information collected from the participants were guaranteed at all times.

### 2.3. Assessment of Emotional and Social Loneliness

This scale is based on the cognitive model of loneliness originally proposed by de Jong Gierveld (1987) [40]. According to this model, the experience of loneliness increases as the discrepancy between desired social relationships and those that one has increases. The de Jong Gierveld Loneliness Scale [41] has been widely used in Europe to measure this construct in the last decade. The scale is based on a robust theoretical model; is simple to apply and brief; and has been validated in multiple countries, showing low susceptibility to cultural biases [42]. The loneliness scale assesses the individual subjective perception of social participation or isolation in the elderly population. Two components are distinguished: emotional loneliness and social loneliness. This questionnaire consists of 11 items, six of which explore emotions linked to the absence of close social connections through negative formulations, thereby evaluating social loneliness. The remaining five items focus on evaluating emotional loneliness [39].

Each item on the scale offers three response options: “no”, “more or less”, and “yes”. Scores are determined from dichotomous responses to the items, where a score of 0 or 1 is assigned according to the instructions given in the instrument. The total score is the sum of the scores for the 11 items, with a minimum possible score of 0 (indicating no loneliness) and a maximum score of 11 (indicating severe loneliness). Three categories based on the score have been established: 0 to 2 points are classified as no loneliness, 3 to 8 points are considered moderate loneliness, and 9 to 11 points are defined as severe loneliness [39].

### 2.4. Psychogeriatric Assessment

The AIS, or Athens Insomnia Scale, is a self-administered assessment instrument used to detect sleep disorders. The complete scale consists of eight items divided into five factors that refer to nighttime sleep and three factors related to daytime dysfunction. These items are scored on a scale ranging from 0 to 24, with the cut-off point set at 6. Higher scores on the scale suggest a greater severity of the problem. It was validated by Soldatos et al. in 2000 [43] and validated in Spanish by Gómez-Benito et al. in 2011 [43].

The Goldberg scale, including anxiety and depression subscales, is both a screening test with healthcare and epidemiological uses and an interview guide. It is a test that not only guides the diagnosis of anxiety or depression (or both in mixed cases) but also discriminates between them and measures their respective intensities [44]. As regards cultural adaptations, the scale has been validated in numerous countries, including the Spanish version [45]. The Goldberg Anxiety and Depression Scale consists of two subscales, each comprising nine questions aimed at evaluating the presence of anxiety (questions 1–9) and depression (questions 10–18). The first four questions of each subscale (questions 1–4 and 10–13, respectively) act as a precondition to determine whether the remaining questions should be answered. Specifically, if at least two questions among questions 1–4 are not answered affirmatively, the remaining questions in the first (anxiety symptoms) subscale need not be answered. On the other hand, in the second (depressive symptoms) subscale, an affirmative answer to one of questions 10 and 13 is sufficient to be able to continue with the remaining questions. We used the score of each subscale in order to separately evaluate depression and anxiety symptoms.

The Barthel Index (BI) was originally conceived to measure the degree of functional autonomy in patients with neuromuscular and musculoskeletal diseases [46]. Although its initial application was in this context, its use has been expanded to various conditions, and it continues to be a commonly used tool in studies on disability in older people, as well as in current clinical practice [47]. The Spanish version, developed by Baztán et al. in 1993 [48], is one of the most widely used instruments to evaluate functional independence in people’s activities of daily living (ADL). It is a way to quantify patients’ ability to perform basic self-care activities [49]. The item scores are added together to obtain a total score ranging from 0 (completely dependent) to 100 (completely independent) [48].

The Lawton and Brody Index was used to measure instrumental activities of daily living (IADL). In 1969, Lawton and Brody developed the Lawton Instrumental Activities of Daily Living Scale (Lawton-IADL) to measure levels of disability and assess parameters in community-dwelling older adults. This scale consists of eight items, including the ability to use the telephone, go shopping, prepare food, do housework, do laundry, use public transportation, manage self-medication, and manage finances. Responses to each of the eight items on the scale are scored as 0 (cannot be performed or can be partially performed) or 1 (can be performed). The total score ranges from 0 (low-functioning, dependent) to 8 (high-functioning, independent). The scale was validated and translated into Spanish by Vergara et al. (2012) [50].

### 2.5. Statistical Analysis

Descriptive statistics were used to describe the quantitative variables, including the mean and standard error of the mean (SEM), as well as range values. Meanwhile, the frequency distribution was presented for the qualitative variables. The normality of the data was verified using the Kolmogorov–Smirnov test. Correlations between the quantitative variables were evaluated using non-parametric Spearman correlation tests. Because the variables did not follow a normal distribution, non-parametric Mann–Whitney U and Kruskal–Wallis tests were used. The proportions were compared using the chi-square test. A logistic and linear regression analysis was also carried out to determine the association between loneliness and the significant variables identified in the previous (bivariate) analyses. A level of *p* < 0.05 was considered statistically significant. All statistical analyses were performed using SPSS 26.0 software (SPSS Inc., Chicago, IL, USA).

## 3. Results

### 3.1. Sample Characteristics of the Study Population

A sample of 108 older people was examined in this study. Most of the participants (66, 61.1%) were women, while 42 were men (38.9%). The average age was 76.09 ± 0.68 (SEM), ranging from 65 to 91 years. As regards marital status, 49 were married (45.4%), 38 were widowed (35.2%), 19 were single (17.6%), and 2 were divorced or separated (1.9%). Their levels of education were as follows: 51 (56.5%) had completed primary education, 18 (16.7%) had completed secondary education, 15 (13.9%) had completed higher education, and 14 (13%) had not completed any level of education. All these data are shown in Table 1, which presents the other sociodemographic variables and psychogeriatric assessments.

### 3.2. Assessment of Loneliness and Its Association with Sociodemographic and Mental Health Factors

The mean emotional loneliness score was 0.79 ± 0.12 (SEM) (range: 0 to 5 points), while the mean social loneliness score was 1.20 ± 0.14 (SEM) (range: 0 to 6 points). The mean loneliness scale score was 2.0 ± 0.23 (SEM) (range: 0 to 10 points). The loneliness variable was categorized using the following cut-off points: 0–2 for the absence of loneliness, 3–8 for moderate loneliness, and 9–11 points for severe loneliness. Consequently, 75 participants (69.4%) did not experience loneliness, while 30 (27.8%) experienced moderate loneliness and 3 (2.8%) showed severe loneliness.

**Figure 1:**
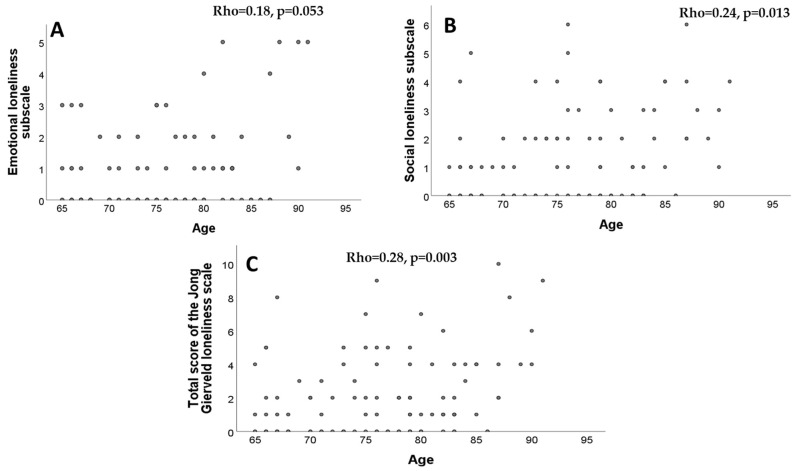
Relationships between age and emotional (**A**), social (**B**), and total (**C**) loneliness on the de Jong Gierveld Loneliness Scale.

No significant correlations were found between the emotional loneliness subscale and age (Rho = 0.18, *p* = 0.051). However, significant correlations were observed between the social loneliness subscale and age (Rho = 0.24, *p* = 0.013), as well as between the total score on the de Jong Gierveld Scale and age (Rho = 0.28, *p* = 0.003; Spearman correlation in cases), as shown in Figure 1.

**Figure 2:**
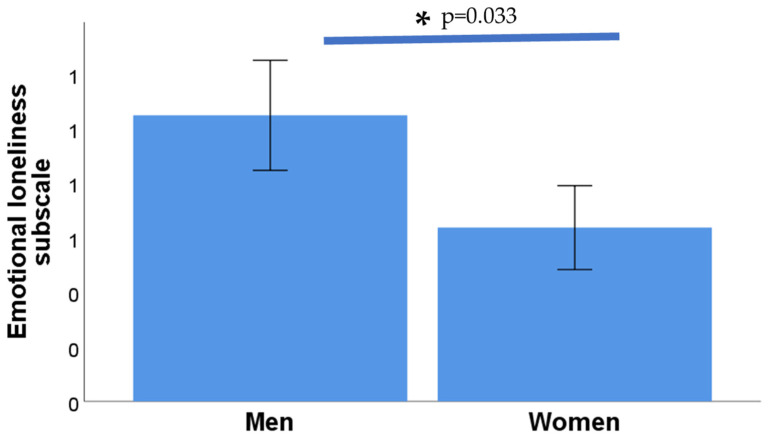
Emotional loneliness scores in men and women. An asterisk indicates a significant difference between the two groups.

On the other hand, significant differences were identified between the emotional loneliness subscale and gender (*p* = 0.033), as shown in Figure 2, but not the social loneliness subscale (*p* = 0.596) or the total score on the de Jong Gierveld Scale (*p* = 0.175; Mann–Whitney U test in all cases).

No significant differences were found between marital status and the emotional loneliness subscale (*p* = 0.674), the social loneliness subscale (*p* = 0.421), or the total score on the de Jong Gierveld Scale (*p* = 0.438; Kruskal–Wallis test in all cases).

No significant differences were found between the emotional and social loneliness subscales or the total score on the loneliness scale and the presence of offspring (*p* = 0.676, *p* = 0.48, and *p* = 0.330, respectively). Likewise, no significant differences were observed between the emotional and social loneliness subscales or total score on the de Jong Gierveld Scale and living with offspring (*p* = 0.18, *p* = 0.567, and *p* = 0.461, respectively; Mann–Whitney U test in all cases).

**Figure 3:**
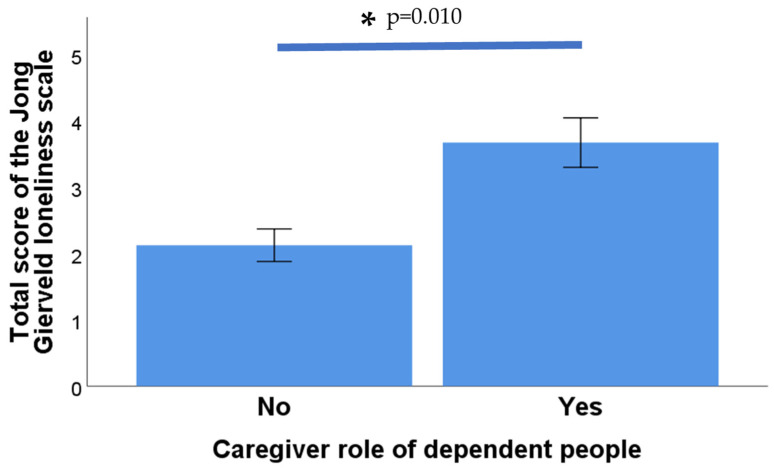
Total loneliness scores in male and female caregivers of dependent people. An asterisk indicates a significant difference between the two groups.

Furthermore, no significant differences were observed between the role of caregiver for a dependent person and the emotional loneliness subscale (*p* = 0.082) or the social loneliness subscale (*p* = 0.094). However, significant differences were found between the total score on the De Jong Gierveld Scale and the role of caregiver for a dependent person (*p* = 0.010; Mann-Whitney U test in all cases), as shown in Figure 3.

Emotional loneliness was significantly associated with poor sleep quality, depressive and anxiety symptoms, basic activities of daily living and instrumental activities of daily living (*p* < 0.05 in all cases, Table 2). Social loneliness was significantly associated with depressive and anxiety symptoms and basic activities of daily living (*p* < 0.05, Table 2).

### 3.3. Multivariate Analyses

Logistic regression analysis was used to explore the associations between the significant variables identified in the bivariate analyses and loneliness, which was defined as a dichotomous variable divided into two categories: no loneliness (0) and moderate/severe loneliness (1). Significant associations were found between loneliness and the presence of offspring (*p* = 0.031; odds ratio [OR] = 0.24; 95% CI 0.06–0.89) and the role of caring for a dependent person (*p* = 0.002; odds ratio [OR] = 0.05; 95% CI 0.009–0.36). However, no associations were identified between loneliness and age (*p* = 0.184; odds ratio [OR] = 1.06; 95% CI 0.97–1.15), gender (*p* = 0.964; odds ratio [OR] = 1.02; 95% CI 0.37–2.77), marital status (*p* = 0.532; odds ratio [OR] = 1.18; 95% CI 0.69–2.00), living with offspring (*p* = 0.493; odds ratio [OR] = 0.63; 95% CI 0.17–2.33), or the presence of grandchildren (*p* = 0.944; odds ratio [OR] = 0.96; 95% CI 0.31–2.99).

Similarly, no significant associations were found between loneliness and sleep quality (*p* = 0.833; odds ratio [OR] = 0.98; 95% CI 0.85–1.13), depressive symptoms (*p* = 0.402; odds ratio [OR] = 1.07; 95% CI 0.91–1.25), the ability to perform basic activities of daily living (*p* = 0.953; odds ratio [OR] = 0.99; 95% CI 0.96–1.03), or the ability to perform instrumental activities of daily living (*p* = 0.394; odds ratio [OR] = 1.43; 95% CI 0.62–3.30).

A linear regression analysis was conducted to explore the relationships between the presence of loneliness as a continuous variable and various predictor variables. These variables included age, gender, marital status, the presence of offspring, living with offspring, caring for a dependent person, sleep quality, depressive symptoms, and the ability to perform basic and instrumental activities of daily living. The results revealed significant associations between the total loneliness score and the role of caring for a dependent person (*p* = 0.002; odds ratio [OR] = 0.34; 95% CI 1.04–4.57). In contrast, no significant associations were found between the total loneliness score and age (*p* = 0.283; odds ratio [OR] = −0.13; 95% CI −0.03–0.13), gender (*p* = 0.082; odds ratio [OR] = −0.18; 95% CI −1.99–0.13), marital status (*p* = 0.203; odds ratio [OR] = 0.18; 95% CI −0.21–1.00), the presence of offspring (*p* = 0.233); odds ratio [OR] = −0.15; 95% CI −2.34–0.58), living with offspring (*p* = 0.771; odds ratio [OR] = 0.03; 95% CI −1.20–1.61), or the presence of grandchildren (*p* = 0.462; odds ratio [OR] = 0.11; 95% CI −0.94–2.06).

Likewise, no significant associations were found between loneliness and sleep quality (*p* = 0.731; odds ratio [OR] = −0.04; 95% CI −0.17–0.12), depressive symptoms (*p* = 0.136; odds ratio [OR] = −0.23; 95% CI −0.04–0.28), the ability to perform basic activities of daily living (*p* = 0.982; odds ratio [OR] = 0.003; 95% CI −0.036–0.037), or the ability to perform instrumental activities of daily life (*p* = 0.678; odds ratio [OR] = −0.04; 95% CI −0.89–0.59).

In addition, linear regression analyses were performed with the emotional and social loneliness subscales. Gender and depressive symptoms were significantly associated with emotional loneliness (Table 3).

On the other hand, no significant associations were identified between the social loneliness subscale and age (*p* = 0.134; odds ratio [OR] = 0.19; 95% CI −0.01–0.10), gender (*p* = 0.434; odds ratio [ OR] = −0.08; 95% CI −0.99–0.42), marital status (*p* = 0.777; odds ratio [OR] = 0.04; 95% CI −0.34–0.46), the presence of children (*p* = 0.479; odds ratio [OR] = −0.09; 95% CI −1.31–0.63), living with a child (*p* = 0.603; odds ratio [OR] = 0.05; 95% CI −0.69–1.18), or the role of caring for a dependent person (*p* = 0.758; odds ratio [OR] = −0.03; 95% CI −1.36–1.00). Likewise, no significant associations were found between the social loneliness subscale and sleep quality (*p* = 0.954; odds ratio [OR] = −0.00; 95% CI −0.10–0.09), depressive symptoms (*p* = 0.323; odds ratio [OR] = 0.16; 95% CI −0.05–0.16), the ability to perform activities of daily living (*p* = 0.634; odds ratio [OR] = 0.05; 95% CI −0.01–0.03), or the ability to perform instrumental activities of daily living (*p* = 0.767; odds ratio [OR] = 0.03; 95% CI −0.42–0.56).

## 4. Discussion

This article is the first attempt to provide detailed data on the prevalence of loneliness in older people living in a rural area of Spain. Our study is the first the analyze the sociodemographic and mental health factors associated with loneliness in older people from *Rincón de Ademuz* in the province of Valencia produced significant results that require meticulous analysis and detailed discussion. In this sample of older people aged between 65 and 91 years from this rural area, we observed that approximately 1 in 3 individuals had experienced some degree of loneliness. Specifically, we found a notable prevalence of moderate loneliness, with 27.8% of participants reporting experiences of social and emotional loneliness. Furthermore, a small but relevant percentage (2.8%) reported feeling severe loneliness. Previous research carried out in urban settings in Spain, namely Valencia and Moncada, which included 530 older adult participants, showed that the prevalence of loneliness was higher than that reported in the present study, with 36.2% of participants experiencing moderate loneliness, while 6.6% reported feeling extreme loneliness.

When analyzing the associations between sociodemographic features and the experience of loneliness, this study examined various factors, such as age, sex, marital status, cohabitation situation, the presence of offspring and grandchildren in the home, and living with a son or daughter. Possible links with depressive symptoms, sleep quality, and the ability to perform basic and instrumental activities of daily living were also investigated. In our study, no significant correlations were found between the emotional loneliness subscale and age. However, significant correlations were observed between the social loneliness subscale and age, as well as between the total score on the de Jong Gierveld Scale and age. While it is true that loneliness can affect people of all ages, there is a general trend that shows an increase in feelings of loneliness as people age, as shown in a study carried out by Gené-Badía et al. in 2020 [51]. This study showed that the level of loneliness is moderately associated with age and that, although loneliness can appear at any age, it is more frequent in older people because they are often widowed or have no partner. One possibility is that the perception of self-isolation can vary with age, in turn influencing the perception of loneliness. A study of older individuals in Malaysia reported that 49.8% of older people are at risk of social isolation [52]. Although an association between age and social isolation has been demonstrated, the perception of loneliness decreases with age, which justifies the results of our study and those of others [53]. In this regard, a meta-analysis showed that there is a U-shaped association, with a lower incidence of loneliness in the middle age ranges and peaks in both the lower and upper ranges among older individuals [4]. 

The relationship between age and loneliness in older people is a complex and multifaceted issue, with multiple factors that contribute to each person’s individual experience [54]. Factors such as changes in social networks over time are common among older people, as they may lose friends and loved ones due to death or physical distance. Those who are single may face a higher risk of loneliness due to a lack of constant company at home, which can reduce their social network and increase the likelihood of feeling lonely [1,54]. Another widely recognized risk factor for loss is the loss of family involvement or a network of friends in the neighborhood, which can occur during the process of downsizing or relocating to a smaller home, condominium, assisted care, or nursing home [54]. Retirement can also lead to the loss of daily interaction with coworkers and friends, contributing to loneliness. Additionally, social activities may become more limited due to mobility or health issues (aging and isolation). Chronic health conditions, such as hearing or vision loss, reduced mobility, and memory loss may make it difficult to participate in social activities and increase feelings of isolation [55]. Age discrimination and stereotypes, manifested as derogatory comments, contribute to the exclusion of older adults from social events, either as a result of not being invited or due to their own reluctance to participate, resulting in their social isolation (aging and isolation).

This study revealed a significant effect of sex regarding the level of loneliness. Although no disparities in social loneliness were observed, men reported experiencing more emotional loneliness than women despite the fact that there were more women in our study. This finding contrasts with previous research that suggested a higher prevalence of loneliness among women than men [3,56]. In 2003, Pinquart and Sörensen concluded that loneliness is associated with several sociodemographic, psychosocial, and health risk factors, including being female, widowed, divorced, or never married; having little contact with important friends or low-quality friendships; deterioration of physical health; and a lack of socioeconomic resources. Similarly, another study reported consistent results, showing a higher prevalence of loneliness among women, participants without a partner, and those who lived alone without offspring [53].

We also sought to explore the impact of the presence of family members on the experience of loneliness in our study. The results of multivariate analyses demonstrated significant associations between loneliness and the presence of offspring but not living with them or the presence of grandchildren (*p* > 0.05). The relationship between having offspring and experiencing loneliness in older people is a complex topic that has been the subject of research in various studies. Some studies suggest that the presence of offspring may act as a protective factor against loneliness in old age, while others have found contradictory results. Some studies have suggested that older people with offspring reported less loneliness, particularly in the emotional realm, while no differences were observed for social loneliness. Having children could, therefore, act as a protective factor against emotional loneliness, but it does not seem to influence individuals’ social life or network of relationships [57]. However, it is important to bear in mind that the presence of offspring does not automatically guarantee fewer experiences of loneliness in old age. The quality of the relationship between parents and offspring, as well as the frequency and nature of the interactions, are important factors to consider. For example, an older person may have offspring who live far away or who have family and work commitments that limit their ability to be present and provide support on a regular basis [58]. Childlessness did not significantly increase the prevalence of loneliness and depression in people with advanced age [58]. There is also no statistical evidence for the hypothesis that childlessness increases loneliness and depression in older people who are divorced, widowed, or have never been married. However, gender alters how childlessness and marital status influence psychological well-being. Divorced, widowed, and never-married men who did not have offspring had significantly higher rates of loneliness compared to women in comparable circumstances [58].

On the other hand, it is important to note that in multivariate analyses, our study found significant associations between the experience of loneliness and the role of acting as a caregiver for a dependent person. Participants who were caregivers reported a greater sense of loneliness compared to those who did not have this responsibility. Becoming a primary caregiver can lead to a reduction in the time available for social interaction, which, in turn, can lead to isolation and a sense of loneliness for the caregiver [54]. Evidence from previous studies has shown that older caregivers of people with dementia reported significantly higher levels of perceived stress, depressive symptomatology, and loneliness compared to non-caregivers who were similar in age, education, global cognition, and functional independence [30]. These people face a considerable emotional and physical burden in caring for their loved ones, which can lead to feelings of isolation and loneliness, especially if they lack adequate social support [27]. Constant dedication to caring for others can limit participation in social and recreational activities, increasing the risk of feeling lonely and disconnected [59]. Additionally, the stress and worry related to caregiving can negatively impact emotional and mental well-being, exacerbating feelings of loneliness. For example, caring for a spouse may result in an isolating experience, with limited opportunities for social engagement. The reported adverse effects of caregiving are diverse and include a decrease in quality of life and emotional and physical problems because of caregiving responsibilities [60]. 

Our study also identified a significant correlation between social and emotional loneliness and depressive symptoms. Although it is not new, this finding reinforces the existing evidence for this relationship. Cacioppo et al. demonstrated a strong association between loneliness and depression in older adults, suggesting that these two factors may act together to reduce well-being in middle-aged and older adults [12]. However, whether loneliness causes depression, whether depression increases feelings of loneliness, or whether both processes occur has not yet been fully established [61]. A longitudinal study conducted in Chicago over a period of 5 years provided solid evidence in this regard by demonstrating that loneliness predicted a subsequent increase in depressive symptomatology but not the other way around [62]. These findings suggest that older people with high levels of loneliness are more likely to experience depressive symptoms [63]. However, the results may vary depending on the cultural and demographic context. For example, a national longitudinal study conducted in Sweden found that increases in depressive symptomatology predicted loneliness in older adults [64].

Cacioppo et al. suggest that the experience of loneliness triggers a series of responses in the brain in order to protect the individual. These include increased vigilance against social threats, sleep problems, activation of the HPA axis, alterations in immune functioning, decreased impulse control, and increased depression [5]. Loneliness has been linked to difficulties falling asleep in older people [65]. One study found that lonely people showed lower levels of sleep efficiency and spent more time awake after falling asleep than non-lonely people [66]. The results suggest that loneliness is associated with more sleep problems and a shorter sleep duration, regardless of various sociodemographic, social network, and health status indicators [67]. The scientific literature has reported the impacts of loneliness on mental health, such as greater loneliness being associated with more fragmented sleep, poorer self-reported sleep quality, and more difficulties sleeping [68,69]. Loneliness has been associated with increased feelings of vulnerability and greater unconscious vigilance against social threats, which contrast with relaxation and deep sleep [5]. In fact, loneliness and low-quality social relationships have been linked to poor sleep quality and daytime dysfunction (e.g., low energy and fatigue) but not sleep duration [66].

The relationship between the ability to perform basic and instrumental activities of daily living (BADL and IADL, respectively) and the risk of loneliness in older people is a topic of great interest in gerontological research. BADL are activities related to basic self-care, such as dressing, bathing, feeding, and getting around [48], while IADL involve more complex tasks of daily life, such as cooking, cleaning, administering medications, performing shopping, and using public transportation [70].

Limitations in the performance of BADL and IADL among older people lead to deterioration of functioning, feelings, and quality of life [71]. Since physical decline is associated with aging and comorbidities, older people inevitably experience limitations in the ability to perform BADL and IADL over time, generating psychological difficulties [72]. These difficulties can restrict participation in social and recreational activities, which, in turn, can lead to feelings of isolation and loneliness [73]. The literature has also shown that factors that may influence associations between perceived accessibility, loneliness, and quality of life include functional disability. For example, sick and disabled older people perceive accessing services and places as more difficult than independent older people [74]. In this case, disabled older adults may find themselves socially isolated, which could also reduce their quality of life [75].

On the other hand, loneliness can also affect an older person’s ability to perform BADL and IADL effectively. The feelings of sadness, lack of motivation, and disinterest that often accompany loneliness can interfere with a person’s ability to care for themselves and perform daily tasks. Additionally, a lack of social support can make it difficult to get help to perform these activities when necessary, which can further exacerbate the situation. A recent study obtained significant results highlighting the relationship between the ability to perform activities of daily living, loneliness, and suicidal ideation in nursing home residents [71]. This study highlights the multifaceted role of social support as a moderator in the influence of limited abilities to perform BADL and loneliness on suicidal ideation among nursing home residents. Furthermore, it was hypothesized that functional disability (IADL limitation scores) and marital status could moderate the indirect pathways between quality of life and feelings of loneliness. The negative relationship between loneliness and quality of life is stronger among elderly people who live without a partner than among those who live with a partner [76]. This indicates that elderly people living without a partner may face a higher risk of poor quality of life, and this risk may increase if they also have lower perceived accessibility to services, greater functional disability, and/or greater feelings of loneliness. This study presents some limitations. For instance, participants were selected by convenience sampling, which could limit the generalization of the results. However, this was a descriptive study with the principal aim of determining the prevalence of loneliness in this population, which was achieved. On the other hand, it was conducted in a concretely rural area. It would be interesting to replicate it in other rural regions, as well as to conduct comparative studies in contexts with different geographic characteristics, such as rural and urban settings. In addition to this, it could be interesting to conduct mixed-methods studies, combining quantitative with qualitative data, to be able to further explain some of the results.

## 5. Conclusions

In conclusion, this study of the prevalence of loneliness in the rural area of *Rincón de Ademuz* shows that approximately one in three older adults experiences feelings of loneliness. Significant correlations were identified between loneliness and sociodemographic variables such as age and gender, and men reported experiencing more emotional loneliness than women, even though more women were included in the sample. The study also found that the presence of offspring acted as a protective factor against loneliness. However, the role of acting as a caregiver for a dependent person was associated with higher levels of loneliness.

Furthermore, relationship were observed between loneliness and depressive symptoms, sleep quality, and the ability to perform basic and instrumental activities of daily living. These findings highlight the complexity of loneliness in older people and the importance of addressing it from multiple perspectives, including sociodemographic, family, and mental health aspects. Taken together, these results highlight the need to implement nurse interventions that address loneliness in older people, considering their individual circumstances and fostering social connection, family support, and access to mental health resources. The main contribution of this study is the exploration of the prevalence of loneliness and associated factors in older adults living in rural areas, an issue that has been understudied so far. Moreover, this research allowed us to identify some of the factors that are related to loneliness perception. This knowledge can contribute to the design of tailored and individualized interventions for this population.

## Figures and Tables

**Table 1:** Sociodemographic characteristics and psychogeriatric assessments.

Variable	Frequency % (Categorical Variables) or Mean ± Standard Error of the Mean (Min–Max Range) (Discrete Variables)
	Sociodemographic characteristics
Age	76.09 ± 0.68 (65–91)
Marital status	
Married	49 (45.4%)
Widowers	38 (35.2%)
Singles	19 (17.6%)
Divorced/separated	2 (1.9%)
Nationality	
Spanish	107 (99.1%)
Foreign	1 (0.9%)
Presence of offspring	
Yes	84 (77.8%)
No	24 (22.2%)
Lives with offspring	
Yes	15 (13.9%)
No	93 (86.1%)
Presence of grandchildren	
Yes	62 (57.4%)
No	46 (42.6%)
	Psychogeriatric assessments
Athens Insomnia Scale	4.94 ± 0.44 (0–21)
Goldberg “depressive symptoms” scale	2.52 ± 0.23 (0–9)
Goldberg “anxiety symptoms” scale	3.39 ± 0.28 (0–9)
Barthel Index (ability to perform basic activities of daily living)	91.14 ± 1.53 (25–100)
Lawton and Brody Index (instrumental activities of daily living)	3.53 ± 0.80 (1–4)

**Table 2:** Relationships between loneliness and insomnia, depressive symptoms, and ability to perform basic and instrumental activities of daily living.

Loneliness Score (De Jong Gierveld Scale Scores)	Variable	Rho and *p* Value
Emotional loneliness subscale	Sleep quality (Athens Insomnia Scale total score)	Rho = 0.22, *p* = 0.012
Social loneliness subscale		Rho = 0.14, *p* = 0.146
Total de Jong Gierveld Scale score		Rho = 0.09, *p* = 0.356
Emotional loneliness subscale	Depressive and anxiety symptoms (Goldberg Scale total score)	Rho = 0.37, *p* = 0.001
Social loneliness subscale		Rho = 0.29, *p* = 0.002
Total de Jong Gierveld Scale score		Rho = 0.25, *p* = 0.007
Emotional loneliness subscale	Basic activities of daily living (Barthel Index total score)	Rho = −0.20, *p* = 0.032
Social loneliness subscale		Rho = −0.22, *p* = 0.027
Total de Jong Gierveld Scale score		Rho = −0.14, *p* = 0.165
Emotional loneliness subscale	Instrumental activities of daily living (Lawton scale total score)	Rho = −0.21, *p* = 0.033
Social loneliness subscale		Rho = −0.06, *p* = 0.536
Total de Jong Gierveld Scale score		Rho = −0.04, *p* = 0.637

**Table 3:** Relationships between emotional loneliness and sociodemographic variables, sleep quality, depressive symptoms, and the ability to perform basic and instrumental activities of daily living.

Variable	*p* Value	OR (Odds Ratio)	CI 95% (Min–Max)
Gender	0.012	−0.26	−1.22–−0.14
Age	0.845	0.02	−0.03–0.04
Marital status	0.063	0.26	−0.01–0.59
Presence of offspring	0.363	−0.11	−1.08–0.40
Living with offspring	0.428	0.08	−0.42–1.00
Cares for a dependent person	0.991	−0.00	−0.90–0.89
Sleep quality	0.462	−0.10	−0.10–0.04
Depressive symptoms	0.048	0.31	−0.001–0.16
Ability to perform basic activities of daily living	0.574	−0.06	−0.02–0.01
Ability to perform instrumental activities of daily living	0.318	−0.11	−0.56–0.18

## Data Availability

Data are contained within the article.

## Public Involvement Statement

No public involvement in any aspect of this research.

## Guidelines and Standards Statement

This manuscript was drafted based on the guidelines for observational studies (STROBE).
